# Unintended pregnancy: magnitude and correlates in six urban sites in Senegal

**DOI:** 10.1186/1742-4755-10-59

**Published:** 2013-11-19

**Authors:** Cheikh Mbacké Faye, Ilene S Speizer, Jean Christophe Fotso, Meghan Corroon, Djimadoum Koumtingue

**Affiliations:** 1 African Population and Health Research Center, Address: APHRC Campus - Manga Close, Off Kirawa Road, P.O. Box 10787–00100, Nairobi, Kenya; 2 University of North Carolina at Chapel Hill, Gillings School of Global Public Health, Department of Maternal and Child Health, Chapel Hill, NC, USA; 3 Concern Worldwide US, New York, USA; 4 IntraHealth International – Senegal Office, Initiative Sénégalaise de Santé Urbaine, Dakar, Senegal

**Keywords:** Pregnancy, Unintended, Urban, Senegal

## Abstract

**Background:**

In Senegal, unintended pregnancy has become a growing concern in public health circles. It has often been described through the press as a sensational subject with emphasis on the multiple infanticide cases as a main consequence, especially among young unmarried girls. Less scientific evidence is known on this topic, as fertility issues are rarely discussed within couples. In a context where urbanization is strong, economic insecurity is persistent and the population is globalizing, it is important to assess the magnitude of unintended pregnancy among urban women and to identify its main determinants.

**Methods:**

Data were collected in 2011 from a representative sample of 9614 women aged 15–49 years in six urban sites in Senegal. For this analysis, we include 5769 women who have ever been pregnant or were pregnant at the time of the survey. These women were asked if their last pregnancy in the last two years was ‘wanted ’then’, ‘wanted later’ or ‘not wanted’. Pregnancy was considered as unintended if the woman responded ‘wanted later’ or ‘not wanted’. Descriptive analyses were performed to measure the magnitude of unintended pregnancies, while multinomial logistic regression models were used to identify factors associated with the occurrence of unintended pregnancy. The analyses were performed using Stata version 12. All results were weighted.

**Results:**

The results show that 14.3% of ever pregnant women reported having a recent unintended pregnancy. The study demonstrates important distinctions between women whose last pregnancy was intended and those whose last pregnancy was unintended. Indeed, this last group is more likely to be poor, from a young age (< 25 years) and multiparous. In addition, it appears that low participation of married women in decision-making within the couple (management of financial resources) and the lack of discussion on family planning issues are associated with greater experience of unintended pregnancy.

**Conclusion:**

This study suggests a need to implement more targeted programs that guarantee access to family planning for all women in need. In urban areas that are characterized by economic insecurity, as in Senegal, it is important to consider strategies for promoting communication within couples on fertility issues.

## Introduction

Increasing attention is being paid to the identification and prevention of unintended pregnancies globally and in sub-Saharan Africa [1,2]. An estimation of the prevalence of unintended pregnancy in sub-Saharan Africa showed that 39% of the 49 million pregnancies in 2008 were unintended, that is, they came earlier than desired or were not wanted at all [1]. There is ample evidence on the negative effects of unplanned pregnancy and fertility on infant, child and mother’s health [3-5], household economic conditions, population growth, and the attainment of the Millennium Development Goals (MDGs) [6]. Unintended pregnancies have been shown to adversely influence maternal and child health seeking behaviors, birth outcomes, and women’s quality of life [7-10]. It has been shown that women who experience an unintended pregnancy are more likely to seek an abortion, which in many cases will be illegal and unsafe in sub-Saharan Africa [3,11]. About a third of unintended pregnancies in sub-Saharan Africa are estimated to end in an abortion [1].

Recent studies in sub-Saharan Africa have been undertaken to examine the extent and determinants of unintended pregnancies. These studies have demonstrated that women experiencing unintended pregnancies are older, more likely to be unmarried, of higher parity, and poorer than women who have not experienced an unintended pregnancy [8,12]. A number of studies on unintended pregnancy prevalence and consequences in sub-Saharan Africa are quantitative [8,12-16], while others include qualitative data collection [13,16,17]. Many of these studies are from rural areas [12,16], from Nigeria [13,15-17] or from Eastern Africa [8,12,14].

The 2010–2011 Senegal Demographic and Health Survey (DHS) indicates that 24.4% of pregnancies in the last five years among women ages 15–49 were considered to be unintended, including 20.4% that came too soon (mistimed) and 4% that was unwanted [18]. Secondary analyses of the 2010–2011 Senegal Demographic and Health Survey indicate that a greater percentage of women in urban areas reported their pregnancy as coming too soon or being unwanted (28.3%), compared to women in rural areas (21.7%) [19].

In the Senegal context, where procreation is only considered socially acceptable within marital unions [20,21], it is not surprising that pregnancies that are experienced outside of union are often considered to be unintended. In particular, estimates from the 2010–2011 Senegal Demographic and Health Survey indicate that 81% of pregnancies in the last five years among unmarried urban women were reported as unintended.

With increased urbanization in sub-Saharan Africa and in Senegal [22], understanding the extent and the determinants of unintended pregnancies is important for ensuring that all women have access to the most effective methods of family planning in order to reduce the occurrence of unintended pregnancies and lower the risks associated with unsafe abortion. With urbanization often come changes in social and sexual norms. In Senegal, a recent study demonstrated that about a third of female urban youth ages 15–24 reported being sexually experienced and among sexually experienced female youth, a third had premarital first sex; these youth are at risk of an unplanned pregnancy [23,24]. Other recent studies on youth in Senegal indicate that unintended pregnancies are often the consequence of a lack of knowledge about reproductive and sexual health as well as a lack of communication between young people and their parents [25].

Given that marriage and childbearing are closely tied in the Senegalese context, rarely has unintended pregnancy been examined among married women; this may be related to fatalistic and pro-natal attitudes in this mostly Muslim population [26]. In the urban context, where it is becoming more expensive to have large families and fertility desires are rapidly declining, studying the magnitude of unintended pregnancy is important for informing future family planning program strategies seeking to target urban Senegalese women (and couples) most in need. This paper seeks to fill these gaps in our understanding about unintended pregnancy using recently collected data from women from urban Senegal. The objectives of this paper are to: a) examine the extent of unintended pregnancy among urban women who have ever been pregnant; b) identify key determinants of unintended pregnancy experience among those women; and c) make programmatic recommendations for improving urban women’s access to and use of family planning to meet current and future fertility desires.

### Methodology

The data from the baseline household survey of the Initiative Sénégalaise de Santé Urbaine (ISSU) were used for this study. This survey was implemented by the Measurement, Learning & Evaluation (MLE) project in 2011. MLE is the evaluation component of the Urban Reproductive Health Initiative established by the Bill and Melinda Gates Foundation in three African countries (Kenya, Nigeria, and Senegal) and in the State of Uttar Pradesh in India.

The data were collected from a representative sample of 9614 women ages 15–49 in six urban areas: Dakar, Pikine, Guédiawaye, Mbao, Kaolack and Mbour. Multi-stage sampling was used to obtain a representative sample of women from each site. In the first stage, enumeration areas were selected in each city with probability proportional to their size; a total of 268 enumeration areas were selected across the six sites. Following a detailed listing of households, twenty-one households were drawn randomly from each selected enumeration area, in the second stage. Finally, all eligible women ages 15–49 years in each household were approached and asked for consent to participate in the survey following completion of a household questionnaire. Prior to the interviews, the household head agreed that the interviewer can approach eligible teenagers to request their participation in the study.

Surveyed women who had a pregnancy in the last two years prior to data collection, were asked if their last pregnancy was desired at that time, wanted later, or was not wanted at all. When the woman was pregnant at the time of the survey, the current pregnancy was considered in the analysis as the last pregnancy. A pregnancy was considered unintended if the women responded: “wanted later” or “not wanted”.

The main outcome variable for this study is the intentionality of the last pregnancy during the two years prior to the survey; this variable was coded as: ‘not pregnant in the last two years’, ‘intended last pregnancy’ and ‘unintended last pregnancy’.

For this analysis of intentionality of the last pregnancy, the study population is women who have ever been pregnant in their life (including those who were pregnant at the time of the survey). The total number of women in the study sample is 5769 in the six study sites.

The main independent variables considered include: education level (coded as none, primary, secondary or higher), religion (Muslims vs. others), marital status (married/in union vs. not married/in union), age (coded as <25, 25–34, 35–39 and 40+),whether the woman worked in the last 12 months (yes vs. no), number of living children (coded as <2, 2, 3, 4+) and ever use of family planning (never used vs. ever used). Other independent variables specific to women in union, such as type of marriage (Polygamous vs. Monogamous), who makes decision on household finances (the wife/jointly coded as 1, husband alone or another person coded as 0 and women who did not work for money coded as 2),who makes decisions on the number of children to have within the couple (the wife/jointly vs. husband alone or another person) and discussion between spouses on family planning within the couple (ever discussed vs. never discussed) are included in the model focused on the married sample. These couple-level variables, reported by women, allow the assessment of a possible influence of partner relations on the pregnancy outcome. Details of these variables are in Table 1. Also included in the analysis is an indicator of economic well-being, the household wealth, calculated as in DHS [27] from households’ assets using principal components analysis. The variable is further recoded into a three equal category variable coded as poor, middle and rich. Final models also control for the urban site using Dakar as the reference group.

**Table 1:** Description of sample of women who have ever been pregnant or are currently pregnant at the time of the survey among women from six urban sites in Senegal, 2011

**Urban site**	**Percent**	**N (Unweighted)**
Dakar	41.3	972
Guédiawaye	10.1	714
Pikine	12.1	705
Mbao	22.4	670
Mbour	6.7	1,305
Kaolack	7.4	1,403
**Age group**		
< 25	19.3	1,171
25 - 34	40.3	2,364
35 - 39	17.7	948
40+	22.7	1,286
**Wealth group**		
Poor	35.7	2,269
Medium	33.1	2,041
Rich	31.2	1,459
**Level of education**		
None	40.8	2,604
Primary	37.0	2,101
Secondary or higher	22.2	1,064
**Religion**		
Muslim	91.5	5,502
Christian and other	8.5	267
**Marital status**		
Not married or in union	17.0	856
Married or in union	83.0	4,913
**Type of marriage**^**a**^		
Monogamous	70.1	3,393
Polygynous	29.9	1,520
**Worked in the last 12 months**		
Did not work	85.6	4,966
Worked	14.4	803
**Who decides about money**		
Husband alone/someone else	57.9	3,331
Wife/husband and wife jointly	42.1	2,438
**Number of living children**		
<2	31.7	1,657
2	20.0	1,163
3	15.4	922
4+	32.9	2,027
**Ever use of a modern method**		
Ever used	60.3	3,301
Never used	38.7	2,420
Don’t know family planning	0.9	48
**Discussion of family planning with spouse**^**a**^		
Ever discussed	58.4	2,843
Never discussed	41.6	2,070
**Decision making on number of children**^**a**^		
Wife/husband and wife jointly	56.8	2,738
Husband alone/someone else	42.3	2,137
Missing	1.0	38
**N**	**100.0**	**5,769**

^a^: Among women in union.

Descriptive analyses were used to assess the level and trends of unintended pregnancies among the study population. Then, multinomial logistic regression analyses were undertaken to identify factors associated with the occurrence of unintended pregnancy, with women reporting unintended pregnancy as the reference group. Three different models were performed to examine a) all women in the sample (Model 1); b) only women in union (Model 2); and c) only women in union, including the couple-level variables (Model 3). All descriptive and multivariate analyses were performed using weights and adjusting for the clustered nature of the data using the svy commands in Stata statistical software version 12. Ethical approval for the study protocol and the informed consent process was obtained from the University of North Carolina at Chapel Hill Institutional Review Board and from the Senegalese Ministry of Health’s National Ethics Committee.

## Results

### Characteristics of the sample

Table 1 shows the sample of women who have ever been pregnant in the six urban sites included. A large proportion of these women are from the Dakar site (41.3%), reflecting the overall distribution of the population across the six sites. The sites of Mbour and Kaolack include low numbers as these are smaller cities (values are 6.7% and 7.4%, respectively). Women included are generally in their prime reproductive years, with about 60% under the age of 35. As expected, about a third of women are in each of the wealth groups. There is also a low level of education with nearly 8 women out of 10 not reaching the secondary level. As is found throughout Senegal, the overwhelming majority (91.5%) of the sample is Muslim. More than 80% of the women who have ever had a birth are in union. This is not surprising in the Senegalese context where marriage and childbearing remain strongly connected [20]. About seventy percent of the women in union are in a monogamous union and 29.9% are in a polygamous union. The sample is mostly composed of unemployed women with 85.6% who have not worked in the last 12 months preceding the survey. The percentage of women who report that they have control over their financial resources is 42.1%. Notably, 48.3% of the women have already had more than 3 children and 60.3% ever used modern family planning including sterilization, intrauterine device, injections, implant, pills, male condom, female condom, emergency contraception, lactational amenorrhea or spermicides. About 6 women in union out of 10 women in union have ever discussed family planning with their spouses and more than half report that the decision on the number of children to have is made by the woman or jointly with her husband/partner.

### Experience of unintended pregnancy by socio-demographic characteristics

As shown in Table 2, half of the ever pregnant women did not have a pregnancy during the past two years prior to the survey. Thirty six percent of the sample reported their last pregnancy in the last two years as intended and the remaining women (14.3%) reported their pregnancy in the last two years as unintended. Across the cities, women from Dakar and Guédiawaye were the least likely to have had a pregnancy in the last two years. The other groups without experience of pregnancy during the last two years, include rich women (55.9%), women over 40 years old (83.9%), Christian women (60.5%), and unmarried women (76.7%). Therefore, more than half of the sample has birth intervals that appear reasonably long (e.g. greater than 2 years, not shown).

**Table 2:** Distribution of ever pregnant women by whether she had a birth/pregnancy since 2009 and the intentionality of the last/current pregnancy by socio-demographic characteristics among women from six urban sites in Senegal, 2011

	**Experience of birth/pregnancy since 2009 and intentionality**			**Total**
	**No pregnancy/birth since 2009**	**Intentional pregnancy/birth**	**Unintentional pregnancy/birth**	
**N (Unweighted)**	**2,829**	**2,029**	**809**	**5,769**
**Percent**	**49.9**	**35.8**	**14.3**	**100.0**
**Urban site**				
Dakar	54.9	33.6	11.6	100.0
Guédiawaye	55.6	29.2	15.3	100.0
Pikine	43.7	36.3	20.0	100.0
Mbao	44.7	40.7	14.6	100.0
Mbour	43.3	39.5	17.2	100.0
Kaolack	46.4	38.4	15.2	100.0
**Age group**				
< 25	24.9	50.4	24.7	100.0
25 - 34	39.1	46.1	14.8	100.0
35 - 39	58.3	28.3	13.4	100.0
40+	83.9	10.9	5.2	100.0
**Wealth group**				
Poor	44.9	35.0	20.1	100.0
Medium	49.6	38.3	12.0	100.0
Rich	55.9	34.0	10.1	100.0
**Level of education**				
None	50.4	35.5	14.1	100.0
Primary	48.6	35.3	16.1	100.0
Secondary or higher	51.3	37.1	11.6	100.0
**Religion**				
Muslim	48.9	36.3	14.8	100.0
Christian and other	60.5	30.6	9.0	100.0
**Marital status**				
Not married or in union	76.7	10.6	12.7	100.0
Married or in union	44.4	41.0	14.6	100.0
**Type of marriage**^**a**^				
Monogamous	39.8	44.5	15.8	100.0
Polygynous	55.3	32.8	11.9	100.0
**Worked in the last 12 months**				
Did not work	49.9	36.1	14.0	100.0
Worked	49.8	34.1	16.1	100.0
**Who decides about money**				
Husband alone/someone else	46.8	37.3	15.9	100.0
Wife/husband and wife jointly	54.2	33.7	12.1	100.0
**Number of living children**				
<2	48.4	40.4	11.1	100.0
2	45.5	41.0	13.5	100.0
3	43.6	39.3	17.1	100.0
4+	57.0	26.5	16.5	100.0
**Ever use of a modern method**				
Ever used	52.2	32.5	15.3	100.0
Never used	46.7	41.2	12.1	100.0
**Discussion of family planning with spouse**^**a**^				
Ever discussed	41.4	41.7	16.9	100.0
Never discussed	48.7	39.9	11.4	100.0
**Decision making on number of children**^**a**^				
Wife/husband and wife jointly	44.8	41.1	14.1	100.0
Husband alone/someone else	44.6	40.0	15.4	100.0

^a^: Among women in union.

Looking at the sub-groups with higher prevalence of unintended pregnancy in the last two years, it is noted that 20.0% of women in Pikine reported their last pregnancy in the last two years as unintended. Likewise, 20.1% of poor women reported an unintended pregnancy in the last two years. Finally, a quarter of the youngest women (<25 years) reported that their last pregnancy in the last two years was unintended.

### Multivariate analyses

The multinomial logistic regression analysis to examine the factors associated with the occurrence of an unintended pregnancy is presented in Table 3 (Models 1 & 2) and Table 4 (Model 3).

**Table 3:** **Multinomial logistic regression relative risk ratios (RRR) and 95**% **CI from analysis of whether ever pregnant women had an intentional or an unintentional pregnancy/birth or no pregnancy/birth since 2009 among all women and then among women in union by socio-demographic characteristics, six urban sites in Senegal, 2011**

**Variables**	**Model 1: All women (Unweighted N = 5,769)**											**Model 2: Women in union (Unweighted N = 4,913)**							
	**Unintended pregnancy/birth vs. No pregnancy/birth since 2009**					**Unintended pregnancy/birth vs. Intended pregnancy/birth**						**Unintended pregnancy/birth vs. No pregnancy/birth since 2009**				**Unintended pregnancy/birth vs. Intended pregnancy/birth**			
	**RRR**		**95% CI**			**RRR**			**95% CI**			**RRR**		**95% CI**		**RRR**		**95% CI**	
**Wealth group [Ref: Poor]**																			
Medium	0.53	^***^	0.40	-	0.71	0.60		^**^	0.45	-	0.80	0.53	^***^	0.41	0.70	0.46	^***^	0.36	0.60
Rich	0.51	^***^	0.36	-	0.72	0.60		^**^	0.43	-	0.84	0.51	^***^	0.35	0.74	0.45	^***^	0.31	0.67
**Level of education [Ref: None]**																			
Primary	1.29	^†^	0.97	-	1.71	1.32		^†^	0.98	-	1.77	1.34	^*^	1.02	1.75	0.88		0.66	1.19
Secondary or higher	1.37		0.92	-	2.05	1.16			0.78	-	1.73	1.39		0.91	2.13	0.78		0.51	1.18
Religion [Ref: Muslim]																			
Christian or other	0.60		0.32	-	1.14	0.62			0.27	-	1.44	0.77		0.39	1.55	0.70		0.30	1.65
**Marital status [Ref: Not married/in union]**																			
Married/in union	1.61	^*^	1.12	-	2.31	0.33		^***^	0.21	-	0.52								
**Age [Ref: < 25 years]**																			
25 – 34	0.11	^***^	0.07	-	0.16	0.42		^***^	0.27	-	0.65	0.17	^***^	0.11	0.26	0.33	^***^	0.21	0.52
35 – 39	0.04	^***^	0.02	-	0.06	0.44		^**^	0.27	-	0.70	0.06	^***^	0.04	0.11	0.40	^***^	0.24	0.67
40+	0.01	^***^	0.00	-	0.01	0.39		^**^	0.21	-	0.73	0.01	^***^	0.01	0.02	0.37	^**^	0.19	0.69
**Work in last 12 months [Ref: Did not work]**																			
Worked	1.15		0.80	-	1.66	1.17			0.84	-	1.65	1.24		0.87	1.78	1.10		0.77	1.57
**Number of living children [Ref: < 2 children]**																			
2	1.99	^**^	1.18	-	3.33	1.80		^*^	1.15	-	2.83	2.02	^*^	1.17	3.48	1.44		0.90	2.30
3	4.66	^***^	2.83	-	7.67	2.82		^***^	1.68	-	4.73	4.39	^***^	2.73	7.04	2.20	^**^	1.36	3.57
4+	7.88	^***^	4.95	-	12.53	4.85		^***^	2.62	-	8.99	8.06	^***^	5.04	12.88	3.82	^***^	2.12	6.89
**Ever use of a method [Ref: Never used]**																			
Ever used	0.71	^*^	0.52	-	0.96	0.65		^*^	0.46	-	0.90	0.80		0.57	1.11	0.47	^***^	0.34	0.66
**Urban Site [Ref: Dakar]**																			
Guédiawaye	1.16		0.80	-	1.69	1.78		^**^	1.24	-	2.54	1.11		0.73	1.69	1.36		0.91	2.03
Pikine	1.85	^**^	1.25	-	2.74	1.66		^**^	1.18	-	2.33	2.05	^***^	1.40	3.02	1.31		0.93	1.83
Mbao	1.16		0.82	-	1.66		1.01		0.63	-	1.61	1.04		0.72	1.52	0.70		0.38	1.27
Mbour	1.19		0.86	-	1.65	1.14			0.82	-	1.58	1.17		0.85	1.62	0.78		0.55	1.10
Kaolack	1.17		0.89	-	1.54	1.15			0.86	-	1.53	1.13		0.86	1.49	0.81		0.60	1.08

^***†***^p < 0.10;^*^p < 0.05; ** p < 0.01; ***p < 0.001.

**Table 4:** **Multinomial logistic regression relative risk ratios (RRR) and 95**% **CI from analysis of whether ever pregnant women in union had an intentional or an unintentional pregnancy/birth or no pregnancy/birth since 2009 by socio-demographic and union characteristics, six urban sites in Senegal, 2011**

**Variables**	**Model 3: Women in union (Unweighted N = 4913)**									
	**Unintended pregnancy/birth vs. No pregnancy/birth since 2009**					**Unintended pregnancy/birth vs. Intended pregnancy/birth**				
	**RRR**		**95% CI**			**RRR**		**95% CI**		
**Wealth group [Ref: Poor]**										
Medium	0.54	^***^	0.41	-	0.71	0.54	^***^	0.41	-	0.70
Rich	0.53	^**^	0.37	-	0.77	0.54	^**^	0.39	-	0.77
**Level of education [Ref: None]**										
Primary	1.35	^*^	1.01	-	1.81	1.04		0.75	-	1.43
Secondary or higher	1.41		0.85	-	2.32	0.95		0.58	-	1.55
**Religion [Ref: Muslim]**										
Christian or other	0.87		0.43	-	1.74	0.74		0.33	-	1.69
**Type of marriage [Ref: Polygamous]**										
Monogamous	1.33		0.88	-	2.01	1.00		0.73	-	1.38
**Age [Ref: < 25 years]**										
25 – 34	0.18	^***^	0.12	-	0.29	0.40	^***^	0.25	-	0.63
35 – 39	0.08	^***^	0.04	-	0.13	0.47	^**^	0.28	-	0.78
40+	0.02	^***^	0.01	-	0.03	0.42	^**^	0.22	-	0.80
**Financial decision making [Ref: Worked for money & Husband only/other decides]**										
Worked for money & Wife/husband and wife jointly decide	0.52	^**^	0.33	-	0.81	0.44	^***^	0.28	-	0.68
Did not work for money	0.87		0.59	-	1.27	0.45	^**^	0.27	-	0.75
**Number of living children [Ref: < 2 children]**										
2	2.22	^*^	1.18	-	4.16	1.85	^*^	1.07	-	3.20
3	4.88	^***^	2.83	-	8.43	2.86	^***^	1.60	-	5.13
4+	9.30	^***^	5.46	-	15.83	4.92	^***^	2.48	-	9.75
**Decision making on number of children [Ref: Husband only/other]**										
Wife/husband and wife jointly	1.00		0.73	-	1.38	1.12		0.81	-	1.54
**Ever use of a method [Ref: Never used]**										
Ever used	1.05		0.68	-	1.61	0.63	^†^	0.40	-	1.02
**Discussion of family planning with spouse [Ref: Never discussed]**										
Ever discussed	0.66	^*^	0.47	-	0.94	0.82		0.58	-	1.16
**Urban Site [Ref: Dakar]**										
Guédiawaye	1.14		0.74	-	1.77	1.57	^*^	1.05	-	2.36
Pikine	2.18	^***^	1.41	-	3.35	1.59	^*^	1.11	-	2.29
Mbao	1.11		0.72	-	1.72	0.84		0.48	-	1.44
Mbour	1.25		0.87	-	1.80	0.94		0.67	-	1.34
Kaolack	1.22		0.88	-	1.69	0.97		0.71	-	1.34

^***†***^p < 0.10;^*^p < 0.05; * p < 0.01; ***p < 0.001.

As shown in Table 3, Model 1 is carried out for all women in the sample. The results show that richer women are less likely to have an unintended pregnancy than to have had no birth in the last two years as compared to poorer women; conversely, they are more likely to be non-pregnant in the last two years than to have experienced an unintended pregnancy. Women aged 25 or older are less likely to have had an unintended pregnancy than to have not gotten pregnant as compared to women under age 25. The opposite pattern is found by number of living children; women with two or more living children are more likely to have had an unintended pregnancy than to have had no pregnancy as compared to women with less than 2 children. Model 1 also includes the comparison between women who had an unintended pregnancy versus women who had an intended pregnancy. As a main result, richer women are less likely to have had unintended pregnancies than intended pregnancies than poorer women. Likewise, women in union and women age 25 and older are also less likely to have had unintended pregnancies than intended pregnancies as compared respectively to unmarried women and women under age 25. Having two or more children is associated with a greater likelihood of having an unintended pregnancy than an intended pregnancy as compared to having fewer than two children. Also, women who ever used a family planning (FP) method are more likely to have had an intended pregnancy than an unintended pregnancy compared to women who never used a FP method. Finally, women living in Guédiawaye and Pikine are more likely to have had an unintended pregnancy than an intended pregnancy.

Model 2 (Table 3) is the same as Model 1 but it is developed only for women in union. Overall, the same pattern for all women (as shown in Model 1) is observed; this is a consequence of the fact that the overwhelming majority of the women in the sample are married or in union. The only difference is that the use of a contraceptive method in the past is only associated with the distinction between having an unintended vs. an intended pregnancy; women who ever used contraception are significantly less likely to have had an unintended than an intended pregnancy.

Table 4 which includes Model 3 is for the same sample as in Model 2 but includes the couple-level variables only asked to women in union. Model 3 shows the same results for the demographic factors as found in Model 2. The examination of the couple-level variables indicates that decision-making about income and discussion of FP between spouses are associated with pregnancy experience and intentionality of the pregnancy. In particular, women who work and are involved in decision-making regarding the management of their own financial income, as compared to women who work and are not involved in decision-making, are less likely to have had an unintended pregnancy. In particular, these women are more likely to have been non-pregnant or to have had an intended pregnancy in the last two years. Further, women who did not work in the last 12 months as compared to women who work and are not involved in financial decision-making are less likely to have had an unintended than an intended pregnancy in the last two years. Finally, women who have discussed family planning with their partner are significantly less likely to have had an unintended pregnancy in the last two years and more likely to have had no pregnancy in the last two years, as compared to women who did not discuss family planning with their partner.

## Discussion

Although previous studies have shown that the risk of unintended pregnancy is higher among unmarried women [21,25], this study demonstrates that married women also experience unintended pregnancies. This result is indicative of unmet needs for family planning among urban women in union and the need to pay greater attention to groups traditionally thought to have lower need for family planning.

This study also demonstrates important distinctions between urban women who have intended pregnancies and those who have unintended pregnancies. Indeed, women with unintended pregnancies are more likely to be poor, from a young age group (< 25 years) and multiparous (have two children or more). Moreover, it appears that a low involvement of married women in decision making within the couple (management of financial resources) and a lack of discussion on FP with the partner are associated with higher experience of unintended pregnancies.

Our findings are similar to those from other studies in sub-Saharan Africa on the extent of unintended pregnancies and factors associated with the occurrence of unintended pregnancies. However, it should be noted that most of these studies are at a national level [28,29] or include only rural samples [13]. The results of our analyses suggest the need to focus on improving the targeting of family planning programs to urban women, particularly urban poor women, as a way to ensure that they can meet their changing fertility desires.

Some socio-demographic factors are not significantly associated with the occurrence of unintended pregnancy. That result was expected given the homogeneity of the sample relatively to the religious group (91.5% of the women are Muslims).

Our findings indicate differences in unintended pregnancy experience across the urban sites included. Women from both Guédiawaye and Pikine experience more unintended pregnancies than women in Dakar, even after controlling for the wealth groups. Given that these sites are both part of the region of Dakar, future studies are needed to better understand ethnic, religion, and behavioral differences in these sites; we also need more information on access to contraception and other health services in these sites. Case studies that include qualitative data collection may be needed to obtain a clearer picture of why these sites are higher risk for unintended pregnancies than the Dakar site.

This study is not without limitations. First, the fact that pregnancies not resulting in a live birth were not taken into account in the study constitutes a source of bias. Those pregnancies that end in abortion are likely to be unintended and thus the true prevalence of unintended pregnancy is likely higher than shown here. In addition, the retrospective question on intentionality of the pregnancy could lead some women to reconsider their responses now that the birth took place. Similarly, because pregnancy intentions are self-reported, women may under or over report unintended pregnancies and there is no way to know the direction of this effect. Finally, the data are cross-sectional and thus it is not possible to know the direction of causality between the variables of interest. For example, while we hypothesize that married women who speak to their spouse are less likely to have unintended pregnancies, the association may be the other way, the experience of an intended (or unintended pregnancy) may lead women (and men) to discuss family planning and future fertility desires. With the data available, it is not possible to know the true direction of causality and thus we discuss associations between these interpersonal variables and experience of an unintended pregnancy. Notably, these limitations should have a minor impact on the scope of the study in view of the large size of the sample and that the main variables of interest are demographic factors associated with experience of an unintended pregnancy.

### Conclusion and recommendations

This study demonstrates that unintended pregnancies in urban Senegal affect both unmarried and married women and the main correlates are parity, age and economic status. In urban areas where non-marital (or pre-marital) sex is becoming more common, and in a setting like Senegal that is predominately Muslim, programs need to consider strategies to get information and counseling to high risk women. This may mean undertaking outreach in poorer urban sites and providing community-based distribution of family planning methods or counseling and referral for women who want methods not available through outreach approaches. In addition, programs can be undertaken to target youth through youth corners in existing health facilities or training providers in offering youth friendly services. Implementation of targeted programmes will guarantee access to family planning for all categories of women in need. In urban areas characterized by economic insecurity, as in Senegal, it is essential to also consider strategies for promoting communication within couples on fertility issues. It is these types of targeted approaches that can help urban women to meet their fertility desires and reduce unintended pregnancies with the overall objective of reducing maternal mortality and morbidity in urban Senegal.

## Competing interests

The authors declare that they have no competing interests.

## Authors’ contributions

CMF conceived of the study, developed the recoded data set, performed the analyses and wrote the initial draft; ISS and JC provided technical guidance at all steps; MC reviewed the tables and DK provided programmatic implications; all authors reviewed and approved the final draft of the paper.

## Authors’ information

Cheikh Mbacké Faye is working with the African Population and Research Center as a Senior Research Officer. He was formerly the Country Manager for the Measurement, Learning and Evaluation project in Senegal. Ilene S Speizer is the Technical Deputy Director for the Measurement, Learning and Evaluation project, University of North Carolina at Chapel Hill – Carolina Population Center, USA. She is also Research Professor in the Department of Maternal and Child Health in the Gillings School of Global Public Health. Jean Christophe Fotso works with Concern Worldwide USA as Associate Director of Research, Monitoring & Evaluation. Formerly, he worked with the African Population and Research Center as Principle Investigator on the Measurement, Learning and Evaluation project. Meghan Corroon is a Technical Officer for the Measurement, Learning and Evaluation project, University of North Carolina at Chapel Hill – Carolina Population Center, USA Djimadoum Koumtingue is working for IntraHealth International (Senegal Office) as a specialist in Monitoring and Evaluation.
